# Downregulation of mapk/mak/mrk overlapping kinase 1 in peripheral blood mononuclear cells of pediatric patients with type 1 diabetes mellitus

**DOI:** 10.5937/jomb0-33220

**Published:** 2022-07-29

**Authors:** Miron Sopić, Ana Ninić, Barbara Ostanek, Dragana Bojanin, Tatjana Milenković, Jelena Munjas, Marija Mihajlović, Jelena Vekić, Janja Marc, Vesna Spasojević-Kalimanovska

**Affiliations:** 1 University of Belgrade, Faculty of Pharmacy, Department of Medical Biochemistry; 2 University of Ljubljana, Faculty of Pharmacy, Department of clinical Biochemistry, Slovenia; 3 Mother and Child Health Care Institute of Serbia "Dr Vukan Čupić", Biochemical Laboratory, Belgrade; 4 Mother and Child Health Care Institute of Serbia "Dr Vukan Čupić", Department of Endocrinology, Belgrade

**Keywords:** type 1 diabetes mellitus, Mammalian target of rapamycin, MAPK/MAK/MRK overlapping kinase 1, dijabetes melitus tip 1, meta rapamicina kod sisara, MAPK/MAK/MRK kinaza 1 koja se preklapa

## Abstract

**Background:**

Type 1 diabetes mellitus (T1DM) is one of the most common endocrine diseases in children. T-cell autoreactivity toward b-cells is controlled by significant changes in metabolism of T cells. Mammalian target of rapamycin (mTOR) is an important intracellular regulator of metabolism and cell growth. MAPK/MAK/MRK overlapping kinase 1 (MOK1) is one of the less known regulators of mTOR. We sought to investigate if MOK1 and mTOR mRNA levels in peripheral blood mononuclear cells (PBMCs) of T1DM pediatric patients are different compared to healthy subjects.

**Methods:**

This study included 172 adolescents with T1DM and 36 healthy adolescent volunteers designated for control group (CG). MOK1 and mTOR mRNA levels were determined in PBMCs by qPCR.

**Results:**

T1DM patients have significant downregulation of MOK1 mRNA levels in PBMCs compared CG (P=0.018), while there was no significant difference in mTOR mRNA levels (P=0.891). Furthermore, in T1DM patients, MOK1 significantly correlated with age, triglycerides and mTOR, while mTOR correlated significantly with BMI and systolic blood pressure. Overweight T1DM subjects had significantly lower MOK1 (P=0.034) and mTOR (P=0.017) mRNA levels, together with significantly higher levels of systolic blood pressure (P<0.001), total cholesterol (P=0.001), LDL-cholesterol (P=0.001) and CRP (P<0.001). Multi - variate analysis showed that MOK1 was independently negatively associated with T1DM when adjusted for sex, age, HDL-C and CRP (OR=0.417 (95%CI: 0.175-0.997), p=0.049).

**Conclusions:**

Our study demonstrated for the first time that T1DM is associated with MOK1 downregulation. In addition, downregulation of both mTOR and MOK1 gene expressions was associated with cardiovascular risk factors in overweight T1DM patients.

## Abstract (sr)

**Uvod:**

Dijabetes melitus tip 1 (T1DM) jedno je od najčešćih endokrinih oboljenja kod dece. Autoreaktivnost T-ćelija prema b-ćelijama kontroliše se značajnim promenama u metabolizmu T ćelija. Cilj delovanja rapamicina kod sisara je važan unutarćelijski regulator metabolizma i rasta ćelija. MAPK/MAK/MRK preklapajuće kinaze koja se preklapa (MOK1) jedan je od manje poznatih regulatora mTOR-a. Cilj istraživanja je bio da se ispita da li su nivoi iRNK MOK1 i mTOR u mononuklearnim ćelijama periferne krvi različiti kod pedijatrijskih pacijenata sa T1DM u odnosu na zdrave ispitanike.

**Metode:**

Ovo istraživanje je obuhvatilo 172 adolescenta sa T1DM i 36 zdravih adolescenata dobrovoljaca koji su činili kontrolnu grupu (CG). Nivoi MOK1 i mTOR mRNA određeni su u PBMC-ima pomoću qPCR-a.

**Rezultati:**

Pacijenti sa T1DM imali su značajno niže nivoe iRNK MOK1 u PBMC u odnosu na CG, dok razlike u nivoima iRNK mTOR nisu bile značajne (P = 0,891). Štaviše, kod pacijenata sa T1DM, MOK1 je značajno korelirao sa godinama, trigliceri dima i mTOR, dok je mTOR značajno korelirao sa BMI i sistolnim krvnim pritiskom. Ispitanici sa prekomernom težinom T1DM imali su značajno niže nivoe iRNK MOK1 (P = 0,034) i mTOR (P = 0,017), zajedno sa značajno većim nivoima sistolnog krvnog pritiska (P <0,001), ukupnog holesterola (P = 0,001), LDL-holesterola (P = 0,001) i CRP (P <0,001). Multivarijantna analiza je pokazala da je MOK1 nezavisno negativno povezan sa T1DM kada je prilagođen polu, starosti, HDL-C i CRP (OR = 0,417 (95%CI: 0,175-0,997), p = 0,049).

**Zaključak:**

Naša studija je prva koja je pokazala da je T1DM udružen sa nishodnom regulacijom MOK1. Pored toga, snižena regulacija ekspresije gena mTOR i MOK1 bila je povezana sa kardiovaskularnim faktorima rizika kod pacije nata sa T1DM sa prekomernom težinom.

## Introduction

Type 1 diabetes mellitus (T1DM) is a chronic disease characterized by dysfunction of pancreatic islet b-cells and concomitant insulin deficiency and hyperglycemia. It is considered as one of the most common endocrine and metabolic diseases in children, and over 500,000 children are currently living with this condition worldwide [1] [2]. Although observed loss of pancreatic islet b-cell's function is currently poorly understood, it is evident that autoreactive T lymphocytes play a critical pathological role in this process [1] [3]. Early stages of T1DM are associated with B cells mediated activation of CD4^+^ and CD8^+^ T cells that lead to selective loss of b-cells [1] [3]. The analysis of Langerhans islets in *post mortem* samples obtained close to T1DM diagnosis showed rare cellular infiltrates dominated by CD4^+^ and CD8^+^ T lymphocytes [4]. It seems that T cell autoreactivity in b-cells autoimmunity is controlled by significant changes in metabolic pathways [5]. Previous studies have shown that these metabolic shifts are highly related to T cells activation, proliferation and differentiation into different subsets, the generation of memory T cells, and the capacity to respond to recall antigen in the long term [5]. Metabolic changes in T cells are even considered as targets for therapy in preclinical models of autoimmunity and transplantation [5]. Mammalian target of rapamycin (mTOR) is considered to be an important intracellular regulator of metabolism and cell growth [5] [6]. This serine/threonine kinase, which is a part of phosphoinositide-3-kinase (PI3K)-AKT pathway, is related to functional regulation of T cells, B cells, neutrophils, macrophages, dendritic cells, mast cells, and natural killer cells [4]. In addition, mTOR seems to be an important player in cytotoxic T cell proteome shaping [7]. mTOR signaling dysfunction has been associated with type 2 diabetes, cancer, neurodegeneration and ageing [6]. One of the less known regulators of mTOR is MAPK/MAK/MRK overlapping kinase1 (also known as renal tumour antigen-1, and serine/threonine kinase 30) (MOK1). It was suggested that MOK1 negatively regulates cilium length of renal epithelial cells by inhibition of mTOR complex 1 (mTORC1) signaling [8]. MOK1 was firstly identified in renal carcinoma cells as an antigen recognized by autologous cytolytic T cells [9]. So far, relatively little is known about MOK1 functions or its upstream and downstream regulators. MOK1 overexpression has been mostly associated with different types of cancer, and this overexpression might be caused by hypomethylation of its promoter [10]. In a recent transcriptome-wide twin study, Huang et al. [11] showed that upregulation of MOK1 in peripheral blood mononuclear cells (PBMC) is associated with hypertension. Principal component analysis of proteins has also revealed MOK1 as relevant to type 2 diabetes mellitus [12].

Considering the fact that 70-90% of PBMCs are T lymphocytes [12], as well as their important role in pathogenesis of T1DM, the main aim of this study was to analyze for the first time whether gene expression levels of MOK1 and mTOR in PBMCs are changed in pediatric patients with T1DM. In order to get a deeper insight into association of these two kinases with other factors tied with T1DM, we sought to investigate if their mRNA levels are related to different demographic, clinical and laboratory characteristics of T1DM patients.

## Materials and methods

This study included 172 adolescents with T1DM (84 males and 88 females) and 36 healthy adolescent volunteers (6 males and 30 females) without family history of T1DM designated for control group (CG). Recruitment of subjects was done in The Mother and Child Health Care Institute of Serbia »Dr Vukan Čupić«, Belgrade, Serbia, during regular follow-up in the outpatient clinic. Criteria from Serbian national guidelines of good clinical practice for diagnosis and treatment of diabetes mellitus were used for diagnosis of diabetes [13] [14]. Tanner scale was used for assessment of puberty stages. The groups were matched by pubertal stage and body mass index (BMI).

All T1DM patients were on insulin therapy. 162 patients were on the intensive insulin therapy regime that included multiple daily insulin injections, and 10 patients were treated with continues subcutaneous insulin infusion through insulin pump. The patients were not on any antihypertensive or lipid-lowering therapy, and there was no clinical nor laboratory evidence of any diabetic complications.

This study was conducted according to guidelines laid down in the Declaration of Helsinki and approved by the Ethics Committees of University of Belgrade-Faculty of Pharmacy and Mother and Child Health Care Institute of Serbia »Dr Vukan Čupić«. All the subjects and their parents were thoroughly informed about all aspects of the study, and written inform consent was obtained.

Whole blood was obtained from all subjects after 12 hours fasting period. Immediately after plasma separation, PBMCs were isolated using density gradient (Ficoll-Paque® PLUS gradient-gel), added to Trizol^TM^ (Invitrogen Life Technologies, Foster City, USA) and stored at -80°C.

Glucose, total cholesterol, HDL-cholesterol, and triglycerides levels were measured in serum using routine laboratory methods. Friedewald formula was used to calculate LDL-cholesterol. HbA1c level was determined by competitive turbidimetric inhibition immunoassay. C-reactive protein (CRP) was measured using immunoturbidimetric method. All the analyses were performed on Roche/Hitachi c501 automated analyzer (Roche, Mannheim, Germany).

Protocols for RNA isolation, reverse transcription and real-time PCR were described elsewhere [15]. In brief, total RNA was isolated using TRIZOL^TM^-chloroform extraction with modified protocol [16]. RNA concentration and contamination for proteins and organic solvents was assessed using UV analysis at 260 nm, 280 nm and 230 nm respectively. Integrity of isolated RNA was checked using electrophoresis on 1% agarose gel.

Reverse transcription was performed on the 7500 Real-Time PCR System (Applied Biosystems, Foster City, CA, USA).

MOK1 gene expression levels were measured by quantitative PCR on 7500 Real-Time PCR System (Applied Biosystems, Foster City, CA, USA) using Taq-Man® 5'-nuclease gene expression assays (Applied Biosystems, Foster City, CA, USA) for MOK1 (Hs00179504_m1) and HOT FIREPol® DNA Polymerase (Solis BioDyne, Tartu, Estonia). MOK1 mRNA levels were normalized to beta-actin (Hs01060665_g1) as a housekeeping gene.

MTOR gene expression levels were measured by quantitative PCR on Lightcycler II (Roche diagnostics) using specific primers (F: 5-AGGCCGCATTGTCTC-TATCAA-3 ; R: 5-GCAGTAAATGCAGGTAGTCATC-CA-3) and 5x HOT FIREPol® EvaGreen® qPCR Super mix (Solis BioDyne, Tartu, Estonia). MTOR mRNA levels were normalized to GAPDH (F: 5-TGCACCACCAACTGCTTAGC-3 ; R: 5-GGCATG-GACTGTGGTCATGAG-3) as housekeeping gene.

### Statistical Analysis

Statistical analysis was performed using a statistical program IBM® SPSS® Statistics version 22 (SPSS Inc., Chicago, USA). Normality of data distribution was tested with Shapiro-Wilk test. Data did not follow normal distribution and were presented as median with interquartile range. Comparisons between the tested groups were done by Mann-Whitney test. Categorical data were given as absolute frequencies and compared by Chi-square test for contingency tables. Associations between clinical data were tested by Spearman's bivariate correlation analysis.

Multivariate binary regression analysis was conducted to determine possible independent association of MOK1 mRNAs and T1DM using data significantly different between tested groups and data which correlate significantly with MOK1 mRNA levels as covariates (sex, age, HDL-C and CRP). Statistically significant p-value was less than 0.05.

## Results

Demographic, clinical and laboratory characteristics of T1DM and healthy controls are presented in the Table 1. The T1DM group had significantly higher percentage of males (P<0.001) and was significantly younger compared to CG (P=0.003). Glucose, HbA1c, CRP and HDL-C levels were significantly higher in T1DM patients (P<0.01, P<0.001 and P=0.009, P=0.001 respectively). In addition, there was no significant difference in systolic blood pressure, total cholesterol, LDL-C and triglycerides levels between observed groups.

**Table 1:** Demographic, clinical and laboratory characteristics of CG and T1DM patients. a – categorical variables (compared with Chi-square test); b – data are presented as median and interquartile range (compared with Mann-Whitney test)

Parameter	CG	T1DM	P
Sex (male/female)^a^	6/30	84/88	<0.001
Age (years)^b^	17 (13–18)	(12–16)	0.003
Tanner stage			0.088
Tanner I	3	28	
Tanner II	1	13	
Tanner III	3	27	
Tanner IV	3	26	
Tanner V	26	81	
Diabetes duration (years)^b^	/	7 (5-8)	
BMI percentiles^b^	54.4 (33.6–78.4)	56.4 (33.4–78.1)	0.831
Systolic blood pressure (mm Hg) ^b^	110 (94–120)	110 (100–115)	0.678
Glucose (mmol/L)^b^	4.79 (4.66–5.13)	10.20 (7.72–13.85)	<0.001
HbA1c (%)	5.2 (4.8–5.0)	7.6 (7.0–8.7)	<0.001
Total cholesterol (mmol/L)^b^	4.01 (3.56–4.46)	3.98 (3.54–4.62)	0.600
HDL-cholesterol (mmol/L)^b^	1.49 (1.26–1.64)	1.63 (1.42–1.88)	0.001
LDL-cholesterol (mmol/L)^b^	2.21 (1.87–2.51)	2.02 (1.68–2.50)	0.246
Triglycerides (mmol/L)^b^	0.81 (0.62–1.03)	0.71 (0.58–0.93)	0.224
CRP (mg/L)^b^	0.30 (0.20–0.50)	0.70 (0.30–1.70)	0.009

**Figure 1:**
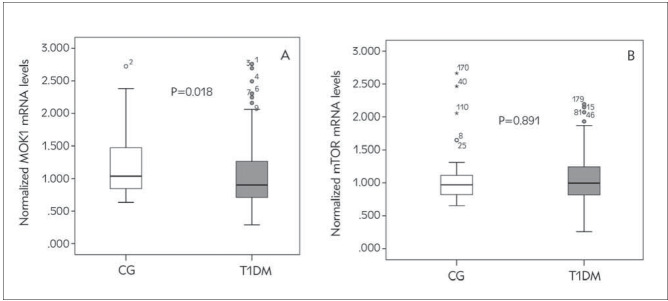
Normalized mRNA levels of MOK1 (A) and mTOR (B) in CG and T1DM patients.

Normalized MOK1 mRNA levels were significantly downregulated in T1DM patients compared to CG (P=0.018) (Figure 1A), while there were no significant differences in normalized mTOR mRNA levels between observed groups (P=0.891) (Figure 1B). MOK1 gene expression levels were not significantly different between males in females in CG (P=0.865), nor in T1DM group (P=0.709). Correlation analysis in T1DM group revealed significant negative correlation of normalized MOK1 mRNA levels with age, and triglycerides and positive association with normalized mTOR mRNA levels, as well as negative association of normalized mTOR mRNA levels with BMI percentiles and systolic blood pressure (Table 2). In addition, we have observed negative trend between BMI percentiles and MOK1 mRNA levels (P=0.091).

**Table 2:** Spearman correlation analysis of MOK1 and mTOR mRNA levels with demographic, clinical and laboratory parameters in T1DM patients.

Parameter	Normalized<br>MOK1 mRNA ρ / P	Normalized<br>mTOR mRNA ρ / P
Age (years)	-0.168 / 0.027	-0.086 / 0.259
BMI percentiles	-0.117 / 0.091	-0.190 / 0.006
Systolic blood pressure (mm Hg)	-0.134 / 0.068	-0.173 / 0.019
Glucose (mmol/L)	-0.080 / 0.302	-0.039 / 0.613
Total cholesterol (mmol/L)	0.005 / 0.945	0.091 / 0.233
HDL-cholesterol (mmol/L)	0.101 / 0.192	0.024 / 0.758
LDL-cholesterol (mmol/L)	-0.016 / 0.841	0.093 / 0.229
Triglycerides (mmol/L)	-0.159 / 0.037	0.010 / 0.892
CRP (mg/L)	-0.066 / 0.397	-0.145 / 0.062
HbA1c (%)	0.051 / 0.502	0.122 /0.111
Normalized MOK1 mRNA	/	0.350 /<0.001
Normalized mTOR mRNA	0.350 /<0.001	/

In order to further explore association between MOK1 and mTOR gene expression with BMI, we have divided T1DM patients according to 85^th^ percentile BMI. Namely, subjects with BMI larger then 85^th^ percentile according to age and gender were considered as overweight. Interestingly, we have observed significant downregulation of MOK1 (P=0.034) and mTOR (P=0.017) in overweight subjects, together with significantly higher levels of systolic blood pressure (P<0.001), total cholesterol (P=0.001), LDL-cholesterol (P=0.001) and CRP (P<0.001). Overweight subjects also had higher triglycerides levels, but this was of borderline significance (P=0.052) (Table 3).

**Table 3:** Demographic, clinical and laboratory characteristics of CG and T1DM patients. a – categorical variables (compared with Chi-square test); b - data are presented as median and interquartile range (compared with Mann-Whitney test)

Parameter	BMI < 85 percentile	BMI 85 percentile	P
Sex (male/female)^a^	69/71	13/14	0.914
Age (years)^b^	14 (12–17)	(13–16)	0.641
Systolic blood pressure (mm Hg)^b^	106 (100–110)	120 (110–120)	<0.001
Glucose (mmol/L)^b^	10.19 (7.74–14.16)	11.59 (7.33–14.04)	0.838
HbA1c (%)	7.6 (6.9–8.6)	7.8 (7.4–9.0)	0.231
Total cholesterol (mmol/L)^b^	3.91 (3.48-4.56)	4.53 (4.14–5.15)	0.001
HDL-cholesterol (mmol/L)^b^	1.64 (1.42–1.89)	1.63 (1.46–1.93)	0.793
LDL-cholesterol (mmol/L)^b^	1.91 (1.62–2.36)	2.48 (2.04–3.08)	0.001
Triglycerides (mmol/L)^b^	0.69 (0.57–0.93)	0.79 (0.67–1.16)	0.052
CRP (mg/L) ^b^	0.60 (0.30–1.20)	1.70 (0.90–3.10)	<0.001
Normalized MOK1 mRNA^b^	0.962 (0.709–1.319)	0.805 (0.654–1.019)	0.034
Normalized mTOR mRNA^b^	1.023 (0.842–1.245)	0.840 (0.769–1.039)	0.017

Binary logistic regression analysis was used to test associations of MOK1 with the presence of T1DM. Univariate analysis revealed significant negative association between MOK1 mRNA and T1DM (OR=0.449 (95%CI: 0.224-0.897), p=0.023). In multivariate analysis, when MOK1 was adjusted for sex, age, HDL-C and CRP, it demonstrated significant independent negative association with T1DM (OR=0.417 (95%CI: 0.175-0.997), p=0.049).

## Discussion

This study, for the first time, demonstrated that MOK1 gene expression levels were downregulated in PBMCs of patients with T1DM compared to healthy controls, whereas there was no significant difference in gene expression level of mTOR between groups. In addition, MOK1 mRNA showed positive correlation with mTOR mRNA, and negative correlation with age, and TG, while mTOR correlated negatively with systolic blood pressure and BMI percentiles.

Although MOK1 was identified 20 years ago as an antigen in renal carcinoma cells that can bind to autologous cytolytic T cells, not much is known about its role in cell signaling [9]. Since its discovery, MOK1 overexpression was observed in numerous types of cancer cells [10]. Quite surprisingly, changes in MOK1 expression levels in PBMCs along with 12 other genes were associated with hypertension in transcriptome-wide twin study [11]. Our study is the first to report that downregulation of MOK1 in PBMCs is associated with T1DM. This association was independent of sex, age, HDL-C and CRP levels. The effects of observed MOK1 downregulation on functions of PBMCs in T1DM are currently not known. One study conducted in cell models for amyotrophic lateral sclerosis suggested that MOK1 inhibition might lead to inflammatory response. Namely, this study showed that aggregates of pathological protein TDP-43 bind to MOK1, disrupting its phosphorylation status supposedly leading to NLRP3/caspase-1 inflammasome activation and secretion of IL-1b and IL-18 [17]. NLRP3/caspase-1 inflammasome activation stimulates secretion of IL-1b and IL-18 [17] [18] and increases systemic inflammation, while IL-1 alone is a major regulator of T-cell proliferation and function leading to polarization of T cells towards proinflammatory immunity [18]. Therefore we can presume that the observed downregulation of MOK1 in PBMCs could lead to increased proinflammatory activity of T cells and contribute to the overall proinflammatory scenery seen in patients with long standing T1DM.

Obesity is considered as a major risk factor for atherosclerosis development and progression and subsequent cardiovascular complications in general population, especially in T1DM patients. It is characterized by proatherogenic lipid profile and increased systemic inflammation [19]. In our study, T1DM patients above the threshold for overweight children (85th percentile BMI according to age and gender), along with higher levels of systolic blood pressure, total cholesterol, LDL-cholesterol, triglycerides and CRP, had significantly lower levels of MOK1 and mTOR gene expressions. Namely, the hormonal changes of normal puberty cause a transient physiologic state of insulin resistance. This insulin resistance is markedly exaggerated in adolescents T1DM, especially in obese ones, leading to defects in both the plasma glucose and lipid-lowering effects of insulin [20]. In addition, peripheral insulin resistance, both in well controlled and poorly controlled T1DM, is reflected by impaired insulin suppression of fatty tissue lipo lysis and lowering of plasma free fatty acids and glycerol levels [20] [21], followed by increased triglycerides levels, which contribute to cardiovascular risk [19] [20]. Therefore, it is not surprising that overweight T1DM patients in our study showed higher levels of proatherogenic lipids (total cholesterol, LDL-cholesterol, triglycerides) compared to non-overweight ones. Along with that, MOK1 was negatively correlated with serum triglycerides, and downregulated in overweight T1DM group, suggesting potential link to higher risk of cardiovascular disease development in T1DM patients.

Furthermore, mTORC1 represents a regulator of cellular nutrient and energy status through stimulation of protein, lipid and nucleotide synthesis [22] [23]. It has been found that attenuation of mTOR signaling in the form of protein complex mTORC1 is associated with increased lipolysis in adipose tissue, as well as with enhanced autophagy in adipocytes of obese patients with T2DM [24]. In addition, it was suggested that the relation between mTORC1 activity and insulin resistance follows a U-shaped curve, where too little or too much mTORC1 activity has a negative impact on systemic metabolism [25]. In that way, downregulation of mTOR seen in overweight T1DM could further aggravate processes leading to peripheral insulin resistance. Taken all together, our findings imply that downregulation of both MOK1 and mTOR genes, together with proatherogenic lipid profile and increased SBP, could contribute to increased risk of atherosclerosis development and cardiovascular complications seen in these patients. However, our conclusions are drawn from different studies that explored function of MOK1 and mTOR in various cell types. Our findings are limited to PBMCs, and require further functional studies to back up our premises.

### Limitations

Our study has several limitations. Firstly, this study was performed on relatively small number of healthy participants. Considering that the participants were pediatric population, we had to take into account the underlying ethical rationale. Next, the percentage of females was significantly higher in CG compared to T1DM group. However, mRNA levels of MOK1 and mTOR were not significantly different between males in females in CG, nor in T1DM group, suggesting that uneven sex distribution between groups did not significantly influence our conclusions. Thirdly, CG was significantly older in comparison to T1DM group. Even though MOK1 exhibited a significant negative correlation with age, we find that this correlation has no significant effect on our conclusions. Namely, our study demonstrated negative association of MOK1 levels and age, and since our CG is older we can presume that this age difference didn't affect the observed difference in MOK1 mRNA levels. Moreover, multivariate binary logistic regression showed that MOK1 is associated with T1DM independently of sex, age, HDL-C and CRP levels.

## Conclusion

Our study demonstrated for the first time that MOK1 downregulation is associated with T1DM, which could lead to increased proinflammatory state in T1DM. In addition, downregulation of both mTOR and MOK1 gene expressions was associated with cardiovascular risk factors in overweight T1DM patients.

## Dodatak

### Acknowledgments

This work was supported by a grant from the Ministry of Education, Science and Technological Development, Republic of Serbia (Project No. 175035), and CEEPUS project (CIII-SI-0611-08-1819) Novel diagnostic and therapeutic approaches to complex genetic disorders. The Authors would like to thank students Nikola Tomić, Olivera Borojev and PhD Klemen Kodrič for help in laboratory work and primers optimization.

### Conflict of interest statement

All the authors declare that they have no conflict of interest in this work.

### List of abbreviations

MOK1, MAPK/MAK/MRK overlapping kinase1;<br>mTOR, Mammalian target of rapamycin;<br>mTORC1, Mammalian target of rapamycin complex 1;<br>NLRP3, NLR family pyrin domain containing 3;<br>PBMC, Peripheral blood mono -nuclear cell;<br>T1DM, Type 1 diabetes mellitus;<br>T2DM, Type 2 diabetes mellitus.

## References

[1] Katsarou A, Gudbjörnsdottir S, Rawshani A, Dabelea D, Bonifacio E, Anderson B J, Jacobsen L M, Schatz D A, Lernmark Å (2017). Type 1 diabetes mellitus. Nature Reviews Disease Primers.

[2] Hashim A A, Ali S A, Emara I A, El-Hefnawy M H (2016). CTX correlation to disease duration and adiponectin in Egyptian children with T1DM. Journal of Medical Biochemistry.

[3] Atkinson M A, Eisenbarth G S, Michels A W (2014). Type 1 diabetes. Lancet.

[4] Roep B O, Peakman M (2011). Diabetogenic T lymphocytes in human Type 1 diabetes. Current Opinion in Immunology.

[5] Bordignon C, Canu A, Dyczko A, Leone S, Monti P (2017). T-cell Metabolism as a Target to Control Autoreactive T Cells in b-Cell Autoimmunity. Current Diabetes Reports.

[6] Liu Y, Zhang D T, Liu X (2015). mTOR Signaling in T Cell Immunity and Autoimmunity. International Reviews of Immunology.

[7] Hukelmann J L, Anderson K E, Sinclair L V, Grzes K M, Murillo A B, Hawkins P T, Stephens L R, Lamond A I, Cantrell D A (2016). The cytotoxic T cell proteome and its shaping by the kinase mTOR. Nature Immunology.

[8] Broekhuis J R, Verhey K J, Jansen G (2014). Regulation of Cilium Length and Intraflagellar Transport by the RCK-Kinases ICK and MOK in Renal Epithelial Cells. PLoS One.

[9] Gaugler B, Brouwenstijn N, Vantomme V, Szikora J P, van der Spek C W, Patard J J, Boon T, Schrier P, van den Eynde B J (1996). A new gene coding for an antigen recognized by autologous cytolytic T lymphocytes on a human renal carcinoma. Immunogenetics.

[10] Qian J, Chen Q, Yao D M, Yang L, Yang J, Wen X M, Zhang Y Y, Chai H Y, Ma J C, Deng Z Q, Lin J (2015). MOK overexpression is associated with promoter hypomethylation in patients with acute myeloid leukemia. International Journal of Clinical and Experimental Pathology.

[11] Huang Y, Ollikainen M, Sipilä P, Mustelin L, Wang X, Su S, Huan T, Levy D, Wilson J, Snieder H, Kaprio J (2018). Genetic and environmental effects on gene expression signatures of blood pressure: A transcriptome-wide twin study. Hypertension.

[12] Bhramaramba R, Rao A A, Kumar V V, Sridhar G R (2005). Principal Component Analysis (Pca) Of Proteins Related To Type 2 Diabetes Mellitus: Comparative. Journal of Theoretical and Applied Information Technology.

[13] 13. Ministry of Health of Republic Serbia (2012). The good clinical practice national guidelines on diabetes mellitus.

[14] Silverstein J, Klingensmith G, Copeland K, Plotnick L, Kaufman F, Laffel L, et al (2005). Care of children and adolescents with type 1 diabetes: A statement of the American Diabetes Association. Diabetes Care.

[15] Sopić M, Joksić J, Spasojević-Kalimanovska V, Kalimanovska-Oštrić D, Anđelković K, Jelić-Ivanović Z (2015). Are decreased AdipoR1 mRNA levels associated with adiponectin resistance in coronary artery disease patients?. Clinical and Experimental Pharmacology and Physiology.

[16] Vujović A, Spasojević-Kalimanovska V, Bogavac-Stanojević N, Spasić S, Kotur-Stevuljević J, Jelic-Ivanović Z (2013). Comparison of two RNA isolation methods for determination of SOD1 and SOD2 gene expression in human blood and mononuclear cells. Indian J Biotechnol.

[17] Leal-Lasarte M M, Franco J M, Labrador-Garrido A, Pozo D, Roodveldt C (2017). Extracellular TDP-43 aggregates target MAPK/MAK/MRK overlapping kinase (MOK) and trigger caspase-3/IL-18 signaling in microglia. FASEB Journal.

[18] Grebe A, Hoss F, Latz E (2018). NLRP3 Inflammasome and the IL-1 Pathway in Atherosclerosis. Circulation Research.

[19] de Ferranti S D, de Boer I H, Fonseca V, Fox C S, Golden S H, Lavie C J, Magge S N, Marx N, Mcguire D K, Orchard T J, Zinman B (2014). Type 1 diabetes mellitus and cardiovascular disease: A scientific statement from the American Heart Association and American Diabetes Association. Circulation.

[20] Priya G, Kalra S (2018). A Review of Insulin Resistance in Type 1 Diabetes: Is There a Place for Adjunctive Metformin?. Diabetes Therapy.

[21] Heptulla R A, Stewart A, Enocksson S, Rife F, Ma T Y, Sherwin R S, Tamborlane W V, Caprio S (2003). In Situ Evidence That Peripheral Insulin Resistance in Adolescents with Poorly Controlled Type 1 Diabetes Is Associated with Impaired Suppression of Lipolysis: A Microdialysis Study. Pediatric Research.

[22] Hosseini M, Nezhadali M, Hedayati M (2021). Association of vaspin rs2236242 gene polymorphism with serum vaspin level, insulin resistance and diabetes in an Iranian diabetic/pre-diabetic population. J Med Biochem.

[23] Saxton R A, Sabatini D M (2017). mTOR Signaling in Growth, Metabolism, and Disease. Cell.

[24] Öst A, Svensson K, Ruishalme I, Brännmark C, Franck N, Krook H, Sandström P, Kjolhede P, Strålfors P (2010). Attenuated mTOR Signaling and Enhanced Autophagy in Adipocytes from Obese Patients with Type 2 Diabetes. Molecular Medicine.

[25] Laplante M, Sabatini D M (2012). mTOR Signaling in Growth Control and Disease. Cell.

